# Association of Japan Coma Scale score on hospital arrival with in-hospital mortality among trauma patients

**DOI:** 10.1186/s12873-019-0282-x

**Published:** 2019-11-06

**Authors:** Tetsuya Yumoto, Hiromichi Naito, Takashi Yorifuji, Toshiyuki Aokage, Noritomo Fujisaki, Atsunori Nakao

**Affiliations:** 1 Department of Emergency, Critical Care, and Disaster Medicine, Okayama University Graduate School of Medicine, Dentistry and Pharmaceutical Sciences, 2-5-1, Shikata-cho, Kita-ku, Okayama, 700-8558 Japan; 2 Department of Epidemiology, Okayama University Graduate School of Medicine, Dentistry and Pharmaceutical Sciences, Okayama, Japan; 3 Department of Geriatric Emergency Medicine, Okayama University Graduate School of Medicine, Dentistry and Pharmaceutical Sciences, Okayama, Japan

**Keywords:** Glasgow coma scale, Japan Coma Scale, Mortality, Trauma, Traumatic brain injury

## Abstract

**Background:**

The Japan Coma Scale (JCS) score has been widely used to assess patients’ consciousness level in Japan. JCS scores are divided into four main categories: alert (0) and one-, two-, and three-digit codes based on an eye response test, each of which has three subcategories. The purpose of this study was to investigate the utility of the JCS score on hospital arrival in predicting outcomes among adult trauma patients.

**Methods:**

Using the Japan Trauma Data Bank, we conducted a nationwide registry-based retrospective cohort study. Patients 16 years old or older directly transported from the trauma scene between January 2004 and December 2017 were included. Our primary outcome was in-hospital mortality. We examined outcome prediction accuracy based on area under the receiver operating characteristic curve (AUROC) and multiple logistic regression analysis with multiple imputation.

**Results:**

A total of 222,540 subjects were included; their in-hospital mortality rate was 7.1% (*n* = 15,860). The 10-point scale JCS and the total sum of Glasgow Coma Scale (GCS) scores demonstrated similar performance, in which the AUROC (95% CIs) showed 0.874 (0.871–0.878) and 0.878 (0.874–0.881), respectively. Multiple logistic regression analysis revealed that the higher the JCS score, the higher the predictability of in-hospital death. When we focused on the simple four-point scale JCS score, the adjusted odds ratio (95% confidence intervals [CIs]) were 2.31 (2.12–2.45), 4.81 (4.42–5.24), and 27.88 (25.74–30.20) in the groups with one-digit, two-digit, and three-digit scores, respectively, with JCS of 0 as a reference category.

**Conclusions:**

JCS score on hospital arrival after trauma would be useful for predicting in-hospital mortality, similar to the GCS score.

## Background

Prompt and accurate assessment of severely injured trauma patients is crucial to guide proper treatment in initial management. To date, the Glasgow Coma Scale (GCS) has been widely utilized to evaluate consciousness level in a variety of clinical settings, including trauma care [1–4]. Triage protocols or outcome prediction models for trauma patients have been developed using the GCS score alone or the GCS score in combination with other clinical variables [5–7]. However, implementing the GCS presents several disadvantages in terms of complexity, essential meaningfulness of the total score, and high inter-rater variability [5, 8, 9].

In Japan, the Japan Coma Scale (JCS) is the most extensively adopted scale for assessing patients’ consciousness level because of its simplicity and applicability [10, 11]. The Japanese Diagnosis Procedure Combination database, which contains nationwide administrative claim and discharge data, includes JCS codes [12–14]. Briefly, the JCS score consists of four main categories: alert (0) and one-, two-, or three-digit codes based on degree of arousal, each of which has three subcategories. Although previous studies have indicated that JCS score correlates with outcomes in stroke patients [10, 15], the JCS has not been examined among trauma patients.

We hypothesized that consciousness level assessed using the JCS score would predict patient outcomes or identify severe traumatic brain injury (TBI) similar to or better than the GCS. A simple assessment tool using the JCS would be useful and pragmatic in the emergency department setting. The aim of this study was to examine the utility of the JCS recorded on hospital arrival in predicting in-hospital mortality in patients after trauma using a large national database, which will help raise worldwide awareness of the JCS score’s applicability.

## Methods

The Okayama University Hospital ethical committee approved the study (ID 1903–021). The requirement for informed consent was waived because patient data was extracted anonymously.

### Study design, setting, and data collection

The design of the present study was a nationwide retrospective cohort study using registry database. We used data from a national trauma registry called the Japan Trauma Data Bank (JTDB), which has been described in detail elsewhere [7, 11]. Briefly, the JTDB was established in 2003 with the Committee for Clinical Care Evaluation of the Japanese Association for Acute Medicine and the Trauma Surgery Committee of the Japanese Association for the Surgery of Trauma. As of March 2018, 272 secondary and tertiary emergency and critical care centers participated in Japanese trauma care and research, from which data patients with Abbreviated Injury Scale (AIS) scores of 3 or above were continuously recorded [16]. The database contains patient demographics, mechanism of injury, vital signs, and consciousness scale ratings based on JCS and the GCS scores on arrival, AIS scores, Injury Severity Score (ISS), treatment, and survival status at hospital discharge. We collected the data from the database, which included data of all trauma patients between 2004 and 2017, provided by the Japan Trauma Care and Research.

### Participants

All patients with trauma who were 16 years old or older directly transported from the injury scene between January 2004 and December 2017 were included in this study. Patients with burn injuries, in cardiac arrest at the scene or on arrival, those with AIS scores of 6 in any region, or those undergoing interfacility transport were excluded.

### Japan Coma Scale

The JCS was first reported in 1974 [17]. The scale is composed of four main categories: 0 and one-, two-, and three-digit codes corresponding with alert, awake without stimuli, arousable with some stimuli (but reverts to previous status if stimulus stops), and unarousable by any forceful stimuli, respectively. Each code is further divided into three subcategories: 1, 2 and 3 in the one-digit code, 10, 20, and 30 in the two-digit code, and 100, 200, and 300 in the three-digit code. Hence, there are 10 grades in total (Additional file 1: Table S1). A JCS score of 0 is equal to a GCS score of 15 (E4V5M6), while a JCS score of 300 corresponds with a GCS score of 3 (E1V1M1).

### Outcome measures

The primary outcome was in-hospital mortality from all causes. The secondary outcome was the presence of severe TBI, which was defined as head AIS scores of 4 or 5.

### Statistical analysis

Continuous variables are presented as median and interquartile range values, whereas categorical variables are shown as frequencies and percentages. To account for missing data, multiple imputation was performed to analyze incomplete data, replacing each missing value with a set of 20 substitute plausible values [18, 19]. Covariables, including sex, mechanism of injury, systolic blood pressure, heart rate, respiratory rate, JCS score, GCS score, ISS, and hospital mortality were used to develop 20 complete data sets. Using the pooled data, we examined ability to accurately predict outcomes based on area under the receiver operating characteristic curve (AUROC) and multiple logistic regression analysis, focusing on JCS and GCS scores. As mentioned above, the JCS score has four main categories with three subcategories except for the JCS score of 0; the simple four JCS categories as well as the 10-point JCS scale were evaluated simultaneously [10]. GCS scores were evaluated using subscores, including those for eye, verbal, and motor responses, in addition to a simple sum score. Adjusted odds ratios (ORs) and their 95% confidence intervals (CIs) for primary outcomes were obtained after adjusting for age (16–39 vs. 40–64 vs. ≥65); gender; mechanism of injury (blunt or other); systolic blood pressure of < 90 mmHg vs. ≥90 mmHg; heart rate of < 120 bpm vs. ≥120 bpm; respiratory rate of ≤9 cpm vs. 10–29 cpm vs. ≥30 cpm; presence or absence of severe TBI (head AIS score of 4 or 5); presence or absence of emergency surgical intervention (craniotomy, thoracotomy, laparotomy, or angioembolization); and ISS score of ≤8 vs. 9–15 vs. ≥16. Adjusted ORs and their 95% CIs for secondary outcome were obtained using the same variables as the primary outcome except for the presence or absence of severe TBI and emergency surgical intervention and ISS. The suggested cut-off value for each score was determined using the Youden index. Complete cases were also assessed using a sensitivity analysis for in-hospital mortality. A two-tailed *P* value of < 0.05 was considered statistically significant. All analyses were conducted using IBM SPSS Statistics 25 (IBM SPSS, Chicago, IL, U.S.A.).

## Results

**Fig. 1:**
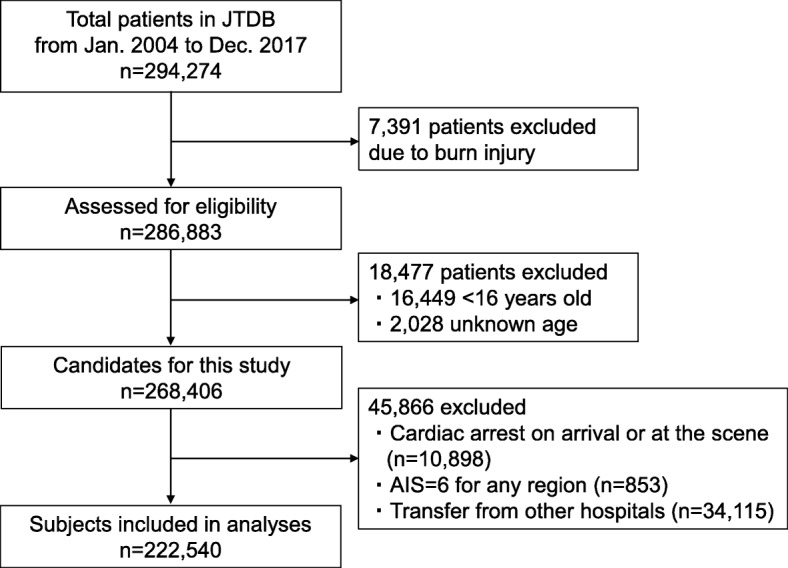
Flow diagram of the study population JTDB: Japan Trauma Data Bank; AIS: Abbreviated Injury Scale.

**Fig. 2:**
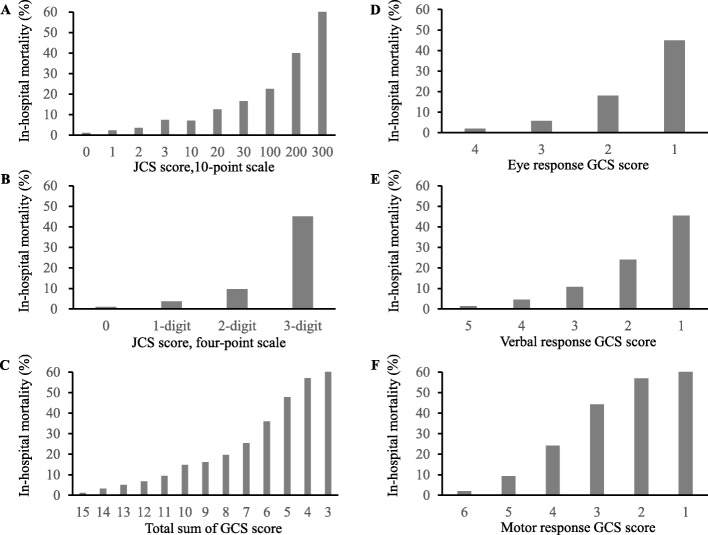
Percentage distribution of in-hospital mortality based on Japan Coma Scale (**a**, **b**) and Glasgow Coma Scale scores (**c**-**f**)

A total of 222,540 trauma patients meeting the inclusion criteria were identified and included in this study (Fig. 1). Study population characteristics are summarized in Table 1. Median age and ISS were 62 and 10, respectively. Of the 222,540 patients, 49,609 (22.3%), 21,388 (9.6%), and 19,424 (8.7%) had missing JCS scores, GCS scores, and in-hospital mortality data, respectively. Complete case demographics are shown in the supplemental material (Additional file 2: Table S2). Regarding consciousness level, 118,158 (53.1%) patients exhibited altered mental status (JCS ≥ 1) according to JCS score. Overall in-hospital mortality was 7.1% (*n* = 15,860). Distribution of in-hospital mortality percentages based on JCS and GCS scores are shown in Fig. 2. The higher the JCS score or the lower the GCS score, the higher the in-hospital mortality rate observed.

**Table 1:** Study population characteristics

	Overall (*n* = 222,540)
Age (years), median (IQR)	62 (41, 77)
16–39 (years), n (%)	53,071 (23.8)
40–64 (years), n (%)	65,572 (29.5)
≥ 65 (years), n (%)	103,897 (46.7)
Male, n (%)	138,023 (62.0)
Blunt mechanism injury, n (%)	213,280 (95.8)
Systolic blood pressure (mmHg), median (IQR)	137 (117, 158)
< 90 mmHg, n (%)	15,672 (7.0)
Heart rate (bpm), median (IQR)	83 (72, 96)
≥120 bpm, n (%)	12,883 (5.8)
Respiratory rate (cpm), median (IQR)	20 (17, 24)
10–29 (cpm), n (%)	196,595 (88.4)
< 10 (cpm), n (%)	3380 (1.5)
≥ 30 (cpm), n (%)	22,565 (10.1)
Japan Coma Scale	
0, n (%)	104,382 (46.9)
1, n (%)	37,934 (17.0)
2, n (%)	21,697 (9.7)
3, (%)	13,929 (6.3)
10, n (%)	14,875 (6.7)
20, n (%)	4320 (1.9)
30, n (%)	3549 (1.6)
100, n (%)	5484 (2.5)
200, n (%)	6810 (3.1)
300, n (%)	9560 (4.3)
One-digit (1, 2, 3), n (%)	73,560 (33.1)
Two-digit (10, 20, 30), n (%)	22,744 (10.2)
Three-digit (100, 200, 300), n (%)	21,854 (9.8)
Glasgow Coma Scale, median (IQR)	15 (13, 15)
≤ 8, n (%)	22,514 (10.1)
Eye response, median (IQR)	4 (3, 4)
Verbal response, median (IQR)	5 (4, 5)
Motor response, median (IQR)	6 (6, 6)
Craniotomy, n (%)	7165 (3.2)
Head AIS score of 4 or 5, n (%)	39,151 (17.6)
Isolated severe TBI, n (%)	9849 (4.4)
Surgical or hemostatic intervention, n (%)	
Thoracotomy, n (%)	2033 (0.9)
Laparotomy, n (%)	5976 (2.7)
Angioembolization, n (%)	5879 (2.6)
Injury Severity Score, median (IQR)	10 (9, 18)
≤ 8, n (%)	42,332 (19.0)
9–15, n (%)	92,531 (41.6)
16≤, n (%)	87,677 (39.4)
In-hospital mortality, n (%)	15,860 (7.1)

*IQR* Interquartile range, *AIS* Abbreviated Injury Scale, *TBI* traumatic brain injury

Data presented in Table 2 shows the performances of both the JCS score and GCS score in predicting in-hospital mortality. AUROC of greater than 0.8 was achieved in both scores. The 10-point scale JCS and the total sum of GCS scores demonstrated similar performance, of which the AUROC (95% CIs) was 0.874 (0.871–0.878) and 0.878 (0.874–0.881), and its suggested cut-off value was 3 on the JCS score and 11 on the GCS score, respectively.

**Table 2:** Predictive performance of the Japan Coma Scale and the Glasgow Coma Scale for in-hospital mortality

	AUROC (95% CI)	Cut-off	Sensitivity, %	Specificity, %	PPV, %	NPV, %
JCS score, 10-point scale	0.874 (0.871–0.878)	3	82.5	78.0	22.4	98.3
JCS score, four-point scale	0.859 (0.856–0.863)	2-digit	76.0	84.3	27.0	97.9
Eye response GCS score	0.829 (0.825–0.833)	2	65.7	92.2	39.3	97.2
Verbal response GCS score	0.860 (0.857–0.864)	3	75.0	87.9	32.2	97.9
Motor response GCS score	0.842 (0.838–0.846)	5	77.5	84.0	27.1	98.0
Total sum of GCS score	0.878 (0.874–0.881)	11	74.7	88.5	33.3	97.9

*AUROC* Area under the receiver operating characteristic curve, *PPV* Positive predictive value, *NPV* Negative predictive value, *JCS* Japan Coma Scale, *GCS* Glasgow Coma Scale

Table 3 shows the results of multiple logistic regression analysis for in-hospital mortality focusing on JCS and GCS scores. The higher the JCS 10-point scale score, the higher the predictability of in-hospital death. When we focused on the simple four-point scale JCS score (0, one-digit, two-digit, and three-digit scores), the adjusted ORs (95% CIs) were 2.31 (2.12–2.45), 4.81 (4.42–5.24), and 27.88 (25.74–30.20) in the groups with one-digit, two-digit, and three-digit scores, respectively, with JCS score of 0 as a reference category. As for GCS score based on the individual component and the total sum of three subscores, lower scores were significantly associated with higher mortality. The eye component showed the highest ability to predict in-hospital death, of which adjusted ORs (95% CIs) were 2.55 (2.54–2.56) for every one unit decrease in the score.

**Table 3:** Multiple logistic regression analysis for in-hospital mortality focusing on the Japan Coma Scale (10-point and four-point scale) and the Glasgow Coma Scale (eye, verbal, and motor response scores and total sum scores) on arrival

	Adjusted ORs	95% CIs	*P*-value
JCS score, 10-point scale			
0	Reference		
1	1.03	0.69–1.53	0.899
2	1.65	1.32–2.01	< 0.001
3	2.19	1.78–2.70	< 0.001
10	3.90	3.15–4.83	< 0.001
20	3.70	2.99–4.57	< 0.001
30	6.26	5.03–7.79	< 0.001
100	8.00	6.26–10.21	< 0.001
200	11.36	9.22–14.00	< 0.001
300	23.95	19.30–29.71	< 0.001
JCS score, four-point scale			
0	Reference		
one-digit	2.31	2.12–2.52	< 0.001
two-digit	4.81	4.42–5.24	< 0.001
three-digit	27.88	25.74–30.20	< 0.001
Eye response GCS score			
E	2.55	2.54–2.56	< 0.001
Verbal response GCS score			
V	2.19	2.18–2.19	< 0.001
Motor response GCS score			
M	2.01	2.00–2.01	< 0.001
Total sum of GCS score			
E + V + M	1.38	1.38–1.38	< 0.001

*ORs* Odds ratios, *CIs* Confidence intervals, *JCS* Japan Coma Scale, *GCS* Glasgow Coma Scale, *TBI* Traumatic brain injury, *ISS* Injury Severity Score

Each adjusted ORs and their 95% CIs were obtained after adjusting for age (16–39 vs. 40–64 vs. ≥65); gender; mechanism of injury (blunt or others); systolic blood pressure of < 90 mmHg vs. ≥90 mmHg; heart rate of < 120 bpm vs. ≥120 bpm; respiratory rate of ≤9 cpm vs. 10–29 cpm vs. ≥30 cpm; presence or absence of severe TBI (head AIS scores of 4 or 5); presence or absence of emergency surgical intervention (craniotomy, thoracotomy, laparotomy, or angioembolization); and ISS of ≤8 vs. 9–15 vs. ≥16. The adjusted ORs of the GCS (eye, verbal, and motor response scores and total sum scores) represent the increase in odds of the outcome with every one unit decrease in the score

As for secondary outcome measures, the 10-point scale JCS score demonstrated the highest predictability for severe TBI, in which the AUROC (95% CIs) was 0.781 (0.778–0.783) with a suggested cut-off value of 2 (Additional file 3: Table S3). In multiple logistic regression analysis, there was a trend toward significance of the association of higher JCS scores with higher predictability of severe TBI (Additional file 4: Table S4). Regarding the GCS score, the eye component showed the highest ability to predict severe TBI, of which adjusted ORs (95% CI) were 2.69 (2.68–2.69) for every one unit decrease in the score.

In sensitivity analysis for in-hospital mortality with complete cases, results were similar to those from the multiple imputation analysis (Additional files 5 and 6: Tables S5 and S6).

## Discussion

In this large Japanese cohort study, we found that assessment of consciousness level based on JCS score at hospital arrival would be useful for predicting in-hospital mortality and severe TBI among adult trauma patients. The JCS score may be similar or even superior to the GCS score in accuracy and simplicity for evaluation of consciousness level.

For decades, the JCS has been used in various emergency settings to assess patients’ consciousness levels in Japan [10, 11, 20–23]. Shigematsu et al. emphasized several advantages of the JCS, including its simplicity, reliability, and applicability [10]. The JCS has four main categories that can be subdivided using the eye response test alone. The GCS is a three-axis scale evaluating eye, verbal, and motor responses, while the JCS is a one-axis scale. Therefore, the same total GCS scores are associated with quite different outcomes, indicating that the total GCS score is meaningless [24]. Our current data showed a correlation between the four-point scale JCS score predictability of in-hospital death and severe TBI. As a two-digit JCS score encompasses E2 and E3 in GCS, the four-point JCS scale would be much simpler, similar to the GCS. Although the 10-point scale JCS assessed verbal or motor responses, it enabled a more detailed evaluation of predictability of in-hospital mortality, as shown in Table 2.

Given the verbal score limitations in intubated patients and complexity of calculating total GCS score, the Glasgow motor scale alone has been investigated for outcome prediction or as a triage test, in which the simplified motor scale has been revealed to have higher predictive power as well as higher interrater reliability [5, 8, 25]. Notably, our results of AUROC> 0.8 demonstrated that either the simplified four-point scale or 10-point scale JCS scores had strong predictive accuracy for in-hospital death, similar to the Glasgow motor scale.

Nonetheless, only a few studies have examined the utility of the JCS score. A previous study showed that simple four-point scale JCS score at onset of stroke significantly correlated with Activities of Daily Living levels at 30 days after the event [10]. Another study described the usefulness of the JCS score in TBI patients in deciding to perform head computed tomography [22]. However, the latter study involved a relatively small sample after mild head injury without evaluating the association between JCS score and clinical outcomes.

To our knowledge, this is the first study to examine the ability of the JCS score to predict mortality and severe TBI among trauma patients. The strength of our study lies in its large cohort from multi-center institutions nationwide, in which we showed the outcome predictability of the 10-point scale JCS as well as the simple four-point JCS. Quick and simple assessment of consciousness level using the JCS will help clinicians identify “red flag” patients immediately.

Our study has several limitations. First, a substantial amount of patient data was missing, including JCS score and mortality. To account for this limitation, multiple imputation was used, in which we confirmed that the same results were obtained as the complete cases. Second, the interrater variation of the JCS score was not evaluated. Although the simple four-point scale JCS could provide consistency among examiners, the 10-point scale might have had variations to some extent as the GCS score had [9, 10]. Nevertheless, the JCS and the GCS assessments were found to be well correlated [22, 26]. Finally, this study was conducted in Japan, where the assessment using JCS has been widespread. An international study should be designed to validate our results.

## Conclusions

In summary, assessment of consciousness level using JCS score on hospital arrival was an efficient predictor of in-hospital mortality among adult trauma patients, similar to the GCS score. This result suggested that the JCS score could be useful and applicable as a triage tool in trauma care settings. Further international studies are warranted to confirm our results.

## Supplementary information

**Additional file 1: Table S1.** Japan Coma Scale scoring.

**Additional file 2: Table S2.** Demographics of complete cases.

**Additional file 3: Table S3.** Predictive performance of the Japan Coma Scale and the Glasgow Coma Scale for severe TBI.

**Additional file 4: Table S4.** Multiple logistic regression analysis for severe traumatic brain injury focusing on Japan Coma Scale score and Glasgow Coma Scale score on arrival.

**Additional file 5: Table S5.** Predictive performance of the Japan Coma Scale and the Glasgow Coma Scale for in-hospital mortality among complete cases.

**Additional file 6: Table S6.** Multiple logistic regression analysis for in-hospital mortality focusing on the Japan Coma Scale (10-point and four-point scale) and the Glasgow Coma Scale (eye, verbal, and motor responses and total sum) on arrival among complete cases.

## Data Availability

The data that support the findings of this study are available from JTDB, but restrictions apply to the availability of these data, which were used under license for the current study, and so are not publicly available. Data are however available from the authors upon reasonable request and with permission of JTDB.

## Abbreviations

AIS: Abbreviated Injury Scale
AUROC: Area under the receiver operating characteristic curve
CIs: Confidence intervals
GCS: Glasgow Coma Scale
ISS: Injury Severity Score
JCS: Japan Coma Scale
JTDB: Japan Trauma Data Bank
ORs: Odds ratios
TBI: Traumatic brain injury
