# Supplementary material for: Association of Japan Coma Scale score on hospital arrival with in-hospital mortality among trauma patients
Source: BMC Emerg Med. 2019 Nov 6;19:65. doi: 10.1186/s12873-019-0282-x (PMC6836363; doi:10.1186/s12873-019-0282-x)
Supplement: Supplementary file 1 — Additional file 1: Table S1. Japan Coma Scale scoring. [file 12873_2019_282_MOESM1_ESM.docx]

**Table S1.** Japan Coma Scale scoring.

| Code | | | Consciousness level |
| --- | --- | --- | --- |
|  |  | 0 | Alert |
| 1-digit | Awake without any stimuli | 1 | Almost fully conscious but not normal |
|  |  | 2 | Unable to recognize time, place, and person |
|  |  | 3 | Unable to recall name or date of birth |
| 2-digit | Arousable by some stimuli but reverts to previous state if stimulus stops | 10 | Arousable by being spoken to |
|  |  | 20 | Arousable by loud voice |
|  |  | 30 | Arousable only by repeated mechanical stimuli |
| 3-digit | Unarousable by any forceful stimuli | 100 | Unarousable but responds to avoid the stimuli |
|  |  | 200 | Unarousable but responds with slight movements, including decerebrate or decorticate postures |
|  |  | 300 | Does not respond at all |
