# Supplementary material for: Association of Japan Coma Scale score on hospital arrival with in-hospital mortality among trauma patients
Source: BMC Emerg Med. 2019 Nov 6;19:65. doi: 10.1186/s12873-019-0282-x (PMC6836363; doi:10.1186/s12873-019-0282-x)
Supplement: Supplementary file 2 — Additional file 2: Table S2. Demographics of complete cases. [file 12873_2019_282_MOESM2_ESM.docx]

**Table S2.** Demographics of complete cases.

| Age (years), median (IQR) | 62 (41, 77) |
| --- | --- |
| 16-39 (years), n (%) | 53,071 (23.8) |
| 40-64 (years), n (%) | 65,572 (29.5) |
| ≥65 (years), n (%) | 103,897 (46.7) |
| Male, n (%) | 137,938 (62.0) |
| Missing, n (%) | 148 (0.1) |
| Blunt mechanism injury, n (%) | 208,666 (93.8) |
| Missing, n (%) | 4,797 (4.2) |
| Systolic blood pressure (mmHg), median (IQR) | 137 (118, 158) |
| <90mmHg, n (%) | 14,096 (6.3) |
| Missing, n (%) | 8,790 (3.9) |
| Heart rate (bpm), median (IQR) | 82 (72, 96) |
| ≥120 bpm, n (%) | 12,243 (5.5) |
| Missing, n (%) | 13,668 (6.5) |
| Respiratory rate (cpm), median (IQR) | 20 (17, 24) |
| 10-29 (cpm), n (%) | 167,056 (75.1) |
| <10 (cpm), n (%) | 1,711 (0.8) |
| ≥30 (cpm), n (%) | 19,497 (8.8) |
| Missing, n (%) | 34,276 (15.4) |
| Japan Coma Scale |  |
| 0, n (%) | 93,467 (42.0) |
| 1, n (%) | 25,068 (11.3) |
| 2, n (%) | 12,466 (5.6) |
| 3, (%) | 8,130 (3.7) |
| 10, n (%) | 11,531 (5.2) |
| 20, n (%) | 2,443 (1.1) |
| 30, n (%) | 2,296 (1.0) |
| 100, n (%) | 4,255 (1.9) |
| 200, n (%) | 5,161 (2.3) |
| 300, n (%) | 8,114 (3.6) |
| one-digit (1, 2, 3), n (%) | 45,664 (20.5) |
| two-digit (10, 20, 30), n (%) | 16,270 (7.3) |
| three-digit (100, 200, 300), n (%) | 17,530 (7.9) |
| Missing, n (%) | 49,609 (22.3) |
| Glasgow Coma Scale, median (IQR) | 15 (14, 15) |
| ≤8, n (%) | 21,081 (9.5) |
| Missing, n (%) | 21,388 (9.6) |
| Eye response, median (IQR) | 4 (3, 4) |
| Missing, n (%) | 20,808 (9.4) |
| Verbal response, median (IQR) | 5 (4, 5) |
| Missing, n (%) | 20981 (9.4) |
| Motor response, median (IQR) | 6 (6, 6) |
| Missing, n (%) | 20,971 (9.4) |
| Craniotomy, n (%) | 7,165 (3.2) |
| Head AIS score of 4 or 5, n (%) | 39,151 (17.6) |
| Isolated severe TBI, n (%) | 9,849 (4.4) |
| Surgical or hemostatic intervention, n (%) |  |
| Thoracotomy, n (%) | 2,033 (0.9) |
| Laparotomy, n (%) | 5,976 (2.7) |
| Angioembolization, n (%) | 5,879 (2.6) |
| Injury Severity Score, median (IQR) | 10 (9, 19) |
| ≤8, n (%) | 39,296 (17.7) |
| 9-15, n (%) | 88,672 (39.8) |
| 16≤, n (%) | 83,621 (37.6) |
| Missing, n (%) | 10,951 (4.9) |
| In-hospital mortality, n (%) | 14,860 (7.3) |
| Missing, n (%) | 19,424 (8.7) |

IQR: ﻿interquartile range; AIS: Abbreviated Injury Scale; TBI: traumatic brain injury.
