# Supplementary material for: Association of Japan Coma Scale score on hospital arrival with in-hospital mortality among trauma patients
Source: BMC Emerg Med. 2019 Nov 6;19:65. doi: 10.1186/s12873-019-0282-x (PMC6836363; doi:10.1186/s12873-019-0282-x)
Supplement: Supplementary file 3 — Additional file 3: Table S3. Predictive performance of the Japan Coma Scale and the Glasgow Coma Scale for severe TBI. [file 12873_2019_282_MOESM3_ESM.docx]

**Table S3**. Predictive performance of the Japan Coma Scale and the Glasgow Coma Scale for severe TBI.

|  | AUROC (95% CI) | Cut-off | Sensitivity, % | Specificity, % | PPV, % | NPV, % |
| --- | --- | --- | --- | --- | --- | --- |
| JCS score, 10-point scale | 0.781 (0.778-0.783) | 2 | 61.8 | 81.3 | 41.3 | 90.9 |
| JCS score, four-point scale | 0.769 (0.767-0.772) | 2-digit | 52.5 | 86.9 | 46.1 | 89.5 |
| Eye response GCS score | 0.713 (0.710-0.716) | 3 | 60.2 | 77.0 | 35.8 | 90.1 |
| Verbal response GCS score | 0.758 (0.755-0.761) | 4 | 72.0 | 72.3 | 35.7 | 92.4 |
| Motor response GCS score | 0.686 (0.683-0.689) | 5 | 49.6 | 85.9 | 42.8 | 88.9 |
| Total sum of GCS score | 0.766 (0.764-0.769) | 14 | 78.2 | 63.7 | 31.5 | 93.2 |

AUROC; area under the receiver operating characteristic curve; PPV: positive predictive value; NPV: negative predictive value; JCS: Japan Coma Scale; GCS: Glasgow Coma Scale.
