# Supplementary material for: Association of Japan Coma Scale score on hospital arrival with in-hospital mortality among trauma patients
Source: BMC Emerg Med. 2019 Nov 6;19:65. doi: 10.1186/s12873-019-0282-x (PMC6836363; doi:10.1186/s12873-019-0282-x)
Supplement: Supplementary file 4 — Additional file 4: Table S4. Multiple logistic regression analysis for severe traumatic brain injury focusing on Japan Coma Scale score and Glasgow Coma Scale score on arrival. [file 12873_2019_282_MOESM4_ESM.docx]

**Table S4.** Multiple logistic regression analysis for severe traumatic brain injury focusing on Japan Coma Scale score and Glasgow Coma Scale score on arrival.

|  | adjusted ORs | 95% CIs | P-value |
| --- | --- | --- | --- |
| JCS score, 10-point scale | | | |
| 0 | Reference |  |  |
| 1 | 0.92 | 0.65-1.31 | 0.639 |
| 2 | 2.55 | 2.19-2.97 | <0.001 |
| 3 | 4.01 | 3.45-4.78 | <0.001 |
| 10 | 6.38 | 5.42-7.52 | <0.001 |
| 20 | 7.22 | 6.12-8.50 | <0.001 |
| 30 | 10.63 | 9.11-12.41 | <0.001 |
| 100 | 16.38 | 13.79-19.45 | <0.001 |
| 200 | 28.69 | 24.09-34.16 | <0.001 |
| 300 | 46.15 | 39.20-54.33 | <0.001 |
| JCS score, four-point scale | | | |
| one-digit | 3.50 | 3.38-3.63 | <0.001 |
| two-digit | 8.62 | 8.26-8.99 | <0.001 |
| three-digit | 37.06 | 35.50-38.69 | <0.001 |
| Eye response GCS score | | | |
| E | 2.69 | 2.68-2.69 | <0.001 |
| Verbal response GCS score | | | |
| V | 2.24 | 2.24-2.24 | <0.001 |
| Motor response GCS score | | | |
| M | 1.90 | 1.90-1.91 | <0.001 |
| Total sum of GCS score | | | |
| E+V+M | 1.38 | 1.38-1.38 | <0.001 |

Each adjusted ORs and their 95% CIs were obtained after adjusting for age (16-39 vs. 40-64 vs. ≥65); gender; mechanism of injury (blunt or others); systolic blood pressure of <90 mmHg vs. ≥90 mmHg; heart rate of <120 bpm vs. ≥120 bpm; and respiratory rate of ≤9 cpm vs. 10-29 cpm vs. ≥30 cpm. The adjusted ORs of the GCS (eye, verbal, and motor response scores and total sum scores) represent the increase in odds of the outcome with every one unit decrease in the score.

ORs: odds ratios; CIs: confidence intervals; JCS: Japan Coma Scale; GCS: Glasgow Coma Scale.
