# Supplementary material for: Association of Japan Coma Scale score on hospital arrival with in-hospital mortality among trauma patients
Source: BMC Emerg Med. 2019 Nov 6;19:65. doi: 10.1186/s12873-019-0282-x (PMC6836363; doi:10.1186/s12873-019-0282-x)
Supplement: Supplementary file 5 — Additional file 5: Table S5. Predictive performance of the Japan Coma Scale and the Glasgow Coma Scale for in-hospital mortality among complete cases. [file 12873_2019_282_MOESM5_ESM.docx]

**Table S5.** Predictive performance of the Japan Coma Scale and the Glasgow Coma Scale for in-hospital mortality among complete cases.

|  | AUROC (95% CI) | Cut-off | Sensitivity, % | Specificity, % | PPV, % | NPV, % |
| --- | --- | --- | --- | --- | --- | --- |
| JCS score, 10-point scale | 0.885 (0.881-0.888) | 3 | 83.2 | 80.0 | 24.4 | 98.4 |
| JCS score, four-point scale | 0.871 (0.867-0.874) | 2-digit | 77.3 | 84.7 | 28.2 | 98.0 |
| Eye response GCS score | 0.830 (0.826-0.834) | 2 | 66.6 | 92.2 | 40.3 | 97.2 |
| Verbal response GCS score | 0.862 80.858-0.866) | 3 | 75.3 | 88.6 | 34.2 | 97.8 |
| Motor response GCS score | 0.840 (0.836-0.845) | 5 | 75.8 | 87.7 | 32.8 | 97.9 |
| Total sum of GCS score | 0.880 (0.877-0.884) | 12 | 77.2 | 87.0 | 31.9 | 98.0 |

AUROC; area under the receiver operating characteristic curve; PPV: positive predictive value; NPV: negative predictive value; JCS: Japan Coma Scale; GCS: Glasgow Coma Scale.
