# Supplementary material for: Association of Japan Coma Scale score on hospital arrival with in-hospital mortality among trauma patients
Source: BMC Emerg Med. 2019 Nov 6;19:65. doi: 10.1186/s12873-019-0282-x (PMC6836363; doi:10.1186/s12873-019-0282-x)
Supplement: Supplementary file 6 — Additional file 6: Table S6. Multiple logistic regression analysis for in-hospital mortality focusing on the Japan Coma Scale (10-point and four-point scale) and the Glasgow Coma Scale (eye, verbal, and motor responses and total sum) on arrival among complete cases. [file 12873_2019_282_MOESM6_ESM.docx]

**Table S6.** Multiple logistic regression analysis for in-hospital mortality focusing on the Japan Coma Scale (10-point and four-point scale) and the Glasgow Coma Scale (eye, verbal, and motor responses and total sum) on arrival among complete cases.

|  | Complete cases | | |
| --- | --- | --- | --- |
|  | adjusted ORs | 95% CIs | P-value |
| JCS score, 10-point scale |  |  |  |
| 0 | Reference |  |  |
| 1 | 1.71 | 1.52-1.92 | <0.001 |
| 2 | 2.07 | 1.82-2.36 | <0.001 |
| 3 | 3.87 | 3.43-4.37 | <0.001 |
| 10 | 3.07 | 2.73-3.45 | <0.001 |
| 20 | 4.56 | 3.84-5.41 | <0.001 |
| 30 | 5.73 | 4.86-6.75 | <0.001 |
| 100 | 9.05 | 7.99-10.24 | <0.001 |
| 200 | 20.52 | 18.38-22.91 | <0.001 |
| 300 | 40.66 | 36.56-45.22 | <0.001 |
| JCS score, four-point scale |  |  |  |
| 0 |  |  |  |
| one-digit | 2.24 | 2.04-2.47 | <0.001 |
| two-digit | 3.61 | 3.25-4.00 | <0.001 |
| three-digit | 21.41 | 19.49-23.53 | <0.001 |
| Eye response GCS score |  |  |  |
| E | 2.27 | 2.22-2.32 | <0.001 |
| Verbal response GCS score |  |  |  |
| V | 2.00 | 1.97-2.03 | <0.001 |
| Motor response GCS score |  |  |  |
| M | 1.84 | 1.81-1.86 | <0.001 |
| Total sum of GCS score |  |  |  |
| E+V+M | 1.33 | 1.32-1.34 | <0.001 |

Each adjusted ORs and their 95% CIs were obtained after adjusting for age (16-39 vs. 40-64 vs. ≥65); gender; mechanism of injury (blunt or others); systolic blood pressure of <90 mmHg vs. ≥90 mmHg; heart rate of <120 bpm vs. ≥120 bpm; respiratory rate of ≤9 cpm vs. 10-29 cpm vs. ≥30 cpm; presence or absence of severe TBI (head AIS score of 4 or 5); presence or absence of emergency surgical intervention (craniotomy, thoracotomy, laparotomy, or angioembolization); and ISS of ≤8 vs. 9-15 vs. ≥16. The adjusted ORs of the GCS (eye, verbal, and motor response scores and total sum scores) represent the increase in odds of the outcome with every one unit decrease in the score.

ORs: odds ratios; CIs: confidence intervals; JCS: Japan Coma Scale; GCS: Glasgow Coma Scale; TBI: traumatic brain injury; ISS: Injury Severity Score.
